# Intraspecific variation reshapes coral assemblages under elevated temperature and acidity

**DOI:** 10.1111/ele.14114

**Published:** 2022-10-09

**Authors:** Mike McWilliam, Joshua S. Madin, Tory J. Chase, Mia O. Hoogenboom, Tom C. L. Bridge

**Affiliations:** ^1^ Hawai'i Institute of Marine Biology University of Hawaiʻi at Mānoa Kāne'ohe Hawaii USA; ^2^ Centre for Biological Diversity, Scottish Oceans Institute University of St Andrews St Andrews UK; ^3^ ARC Centre of Excellence for Coral Reef Studies James Cook University Townsville Queensland Australia; ^4^ College of Science and Engineering James Cook University Townsville Queensland Australia; ^5^ Department of Geography and the Environment Villanova University Villanova Pennsylvania USA; ^6^ Biodiversity and Geosciences Program Museum of Tropical Queensland, Queensland Museum Townsville Queensland Australia

**Keywords:** adaptation, community assembly, coral reefs, functional traits, phenotypic plasticity, physiology

## Abstract

Insights into assemblages that can persist in extreme environments are still emerging. Ocean warming and acidification select against species with low physiological tolerance (trait‐based ‘filtering’). However, intraspecific trait variation can promote species adaptation and persistence, with potentially large effects on assemblage structure. By sampling nine coral traits (four morphological, four tissue and one skeletal) along an offshore–inshore gradient in temperature and pH, we show that distantly related coral species undergo consistent intraspecific changes as they cross into warm, acidic environments. Intraspecific variation and species turnover each favoured colonies with greater tissue biomass, higher symbiont densities and reduced skeletal investments, indicating strong filtering on colony physiology within and across species. Physiological tissue traits were highly variable within species and were independent of morphology, enabling morphologically diverse species to cross into sites of elevated temperature and acidity. Widespread intraspecific change can therefore counter the loss of biodiversity and morphological structure across a steep environmental gradient.

## Graphical abstract

Our results show that distantly related coral species from different families change their traits in a consistent and predictable way across warming, acidifying gradient. We argue that these changes allow an unexpectedly large range of corals to persist in a warm, acidic environment, thereby helping to maintain diversity.

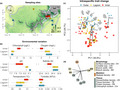

## INTRODUCTION

Marine ecosystems are in transition as populations and communities respond to global warming and ocean acidification (Poloczanska et al., 2013; Smale et al., 2019). Observations of naturally extreme ocean conditions in which temperature and pH levels go beyond typical present‐day ranges suggest that ecological change could reach an endpoint in which key ecosystem functions rely almost entirely on highly tolerant species (Brandl et al., 2020; Fabricius et al., 2011). However, despite extensive research on the types of species likely to persist under new temperature and pH regimes, our understanding of how and why species respond differently to extreme environmental conditions is still emerging, and our capacity to project ecological dynamics remains limited (Urban et al., 2016).

Species traits related to nutrient and resource economics may be useful to understand how community structure can adjust to the extreme environments (Pörtner & Farrell, 2008; Somero, 2010). A range of physiological traits (e.g. in plants: leaf mass per area, leaf dry matter content, leaf nitrogen) can be used to describe integrated responses to stress (Chapin III et al., 1993) and reveal different resource‐use strategies across species (Westoby et al., 2002). Close associations of physiological traits with temperature and precipitation along latitudinal and elevation gradients (Wright et al., 2005), and through time (Li et al., 2015; Soudzilovskaia et al., 2013), indicate that climate change can act as an abiotic ‘filter’ in which only species with well‐adapted traits can persist. Nevertheless, in the long term, a range of structural and physiological traits are likely to interact with multiple abiotic variables to determine community responses to climate variation (Bjorkman et al., 2018; Kühn et al., 2021). Consequently, the influence of traits on ecological dynamics in novel or extreme environments is unresolved.

At the scale of local species assemblages, a large amount of physiological trait variation is explained by differences among individuals of the same species (Messier et al., 2010). ‘Intraspecific variation’ (genetic differences and phenotypic plasticity within a species) can generate wider niche widths, allowing species to occur more broadly across environmental gradients (Benito Garzón et al., 2011; Sides et al., 2014), and genetic components can provide raw materials for evolutionary adaptation (Somero, 2010). Environmental filtering acts both within and across species, selecting individuals with well‐adapted traits or high phenotypic plasticity (Violle et al., 2012). Consequently, when environmental filtering occurs, intraspecific variation allows species to adjust their trait values to match that of the emergent community, allowing more species to persist than expected based on mean trait values alone (Jung et al., 2010). Quantifying the direction and magnitude of the within‐species variation is therefore a key priority to identify traits that predict survival in a changing environment and is crucial to anticipate the scale of species turnover in communities.

For reef‐building corals, warmer and more acidic conditions can alter the dynamics between the host and symbiont (Morris et al., 2019; Muscatine et al., 1984; Tremblay et al., 2014). Most notably, the increasing incidence of marine heatwaves has triggered frequent coral bleaching events, where rapid declines in symbiont numbers lead to reduced health and widespread mortality in the coral host (Hughes et al., 2018). Longer‐term exposure to a warm or thermally variable environment can have more varied effects on host–symbiont relationships, including shifts in the taxonomic identity of symbionts (Fabricius et al., 2004), altered host–symbiont nutritional dynamics (Hoadley et al., 2021), reduced calcification (Crook et al., 2013; Strahl et al., 2015) and higher thermal tolerance of certain species (Oliver & Palumbi, 2011; Wall et al., 2021). While warming and acidification disproportionally impact species with three‐dimensional, branching morphologies (Fabricius et al., 2004; Hughes et al., 2018), long‐term, intraspecific change can occur across a range of morphological types (Guest et al., 2012) and could therefore have large impacts on reef composition and function under changing conditions.

**FIGURE 1:**
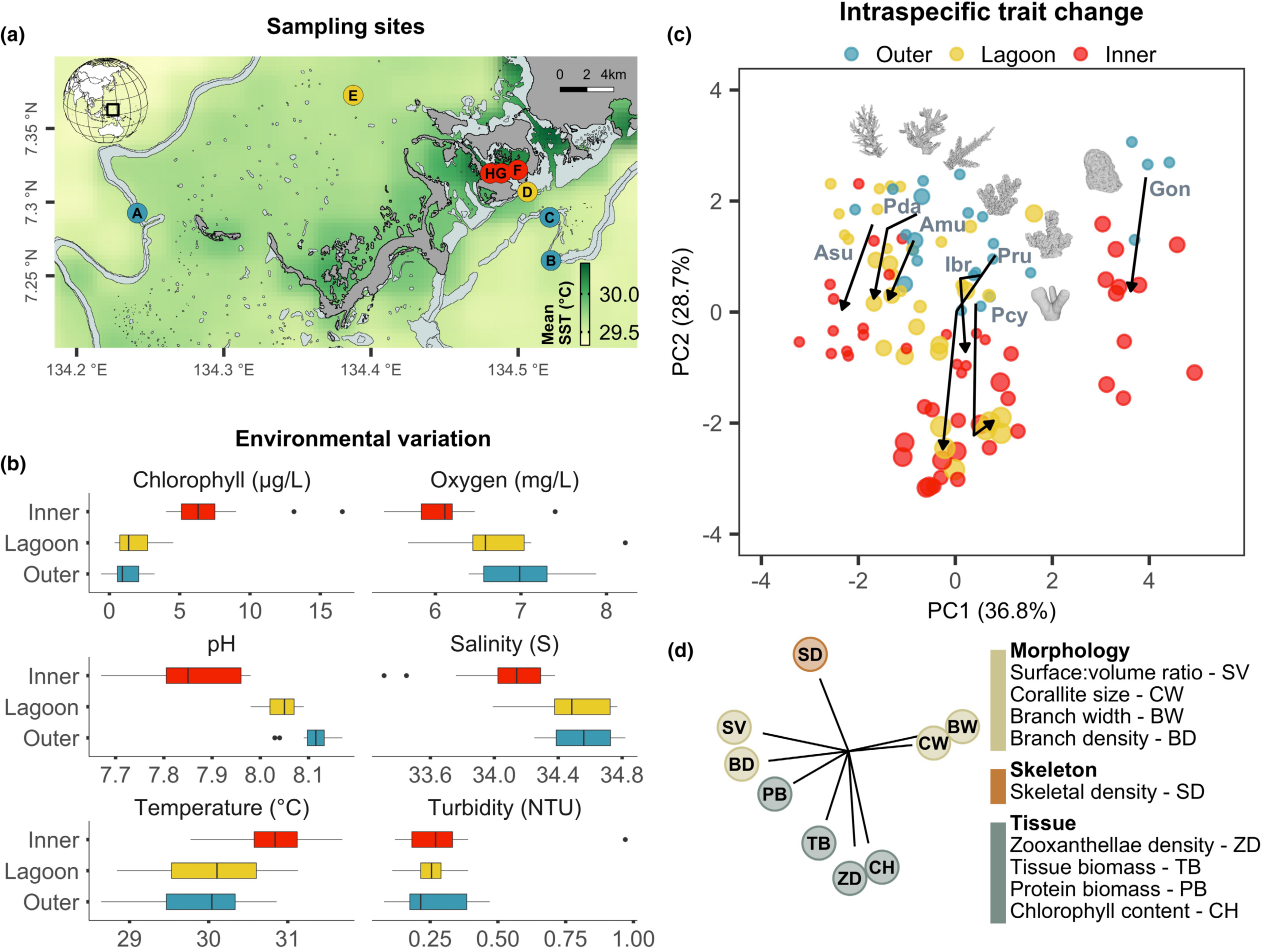
Intraspecific trait change along a steep environmental gradient. (a) Eight sampling locations from outside to inside the Rock Islands in Palau. The green colour gradient indicates decadal SST averages from NOAAs coastal watch program. (b) In situ measurements of environmental variables across the outer barrier, lagoon and inner Rock islands at each sampling site. Measurements were taken on four replicate days within the same 4–5 h window. (c) Principal component analysis (PCA) of sampled colonies (points) by nine traits for the seven species that crossed the gradient. Arrows indicate mean trait shifts between the outer, lagoon and inner sites for each species. Point size is proportional to species relative abundance (Asu = *Acropora* cf. *subglabra*, Pda = *Pocillopora* cf. *damicornis*, Amu = *Acropora* cf. *muricata*, Ibr = *Isopora* cf. *brueggemanni*, Pru = *Porites* cf. *rus*, Pcy = *Porites* cf. *cylindrica*, Gon = *Goniastrea* sp.). (d) The nine trait vectors of the PCA, coloured by trait type (morphology, tissue or skeleton).

Quantifying the importance of intraspecific change for assemblage dynamics remains a key challenge on coral reefs. We measured species and assemblage‐level variation in nine coral traits related to energy acquisition and allocation and analysed how they change along a steep environmental gradient in Palau (Figure 1a,b). The gradient encompasses a range of environmental conditions, from clear, oceanic water considered ‘optimal’ for present‐day coral reefs, to high‐temperature, low‐pH bays, with comparable acidity levels to those projected for the year 2100 (Golbuu et al., 2016; Shamberger et al., 2014). Coral assemblages across the gradient vary in composition, but maintain similar levels of coral cover and taxonomic diversity (Barkley et al., 2015), and foster bleaching‐tolerant populations in high‐temperature locations (Rivera et al., 2022; van Woesik et al., 2012). We aimed to find out how intra‐ and interspecific variation in physiological and structural traits influence assemblage composition under naturally elevated temperature and acidity and ultimately generate new knowledge of the impact of intraspecific variation on species persistence and assemblage dynamics across a steep, environmental gradient.

## MATERIALS AND METHODS

### Study sites and sampling

We collected coral trait and abundance data along an environmental gradient occurring from inside to outside the Rock Islands in Palau (Figure 1a, 7°20′3″N, 134°27′5″E). Coral assemblages were examined at eight sites occurring only a few kilometres apart (Table S1), yet spanning a wide range of environmental conditions (Barkley et al., 2015; Colin, 2018; Golbuu et al., 2016). At each site, six environmental variables were measured locally (Figure 1b), with sensor deployments at each site within the same 4–5 h window (typically 9:00–14:00 h), and replicated across 4 days (see Supplementary Methods for details of instruments and techniques). Three sites were located inside the Rock Islands, with consistently high temperature (Colin, 2018), and low pH (presented on an NBS scale), in addition to higher chlorophyll content, lower salinity and lower dissolved oxygen (Figure 1a,b). Five sites were located outside the Rock Islands (two in the Palau lagoon, and three outer barrier sites exposed to oceanic water), which had lower temperatures and higher pH (Figure 1a,b), and therefore occurred under typical, present‐day seawater conditions considered optimal for coral reefs. To define the area of sampling at each site, we used three circular photomosaics, each 110 m^2^ in the area and adjoining the reef crest at 1–2 m depth (Pizarro et al., 2017). Replicate plots were haphazardly placed 20–60 m apart within equivalent shallow reef crest habitat. The sampling of coral traits and abundance at each site occurred exclusively within the replicate plots. All quantitative analyses were performed on samples pooled into ‘inner’ versus ‘outer’ sites, where the outer includes both barrier and lagoon reefs. However, lagoon reefs are visualised separately in some figures to highlight gradual changes along the gradient (Figure 1a,b).

Following preliminary surveys of each reef, we identified 16 coral species for sampling of which seven were habitat ‘generalists’ that crossed the gradient from the outer reefs into the rock islands, and nine were habitat ‘specialists’ that did not cross the gradient but were instead locally dominant at either outer or inner reef sites. Species chosen encompassed a wide range of growth forms and represented both the ‘robust’ and ‘complex’ clades of the scleractinian phylogeny (Huang, 2012). For each species, we selected 2–4 colonies at each of the reefs where they occurred, took planar and perpendicular images with a scalebar for morphological analysis and sampled one 5–10 cm diameter fragment (a branch or a lobe depending on morphology) for physiological analysis and taxonomic identification. Fragments were immediately frozen in liquid nitrogen for storage and transportation. Specimens were identified based on colony morphology with reference to the type material and original descriptions of each nominal species, combined with recent morpho‐molecular revisions of relevant genera (see Supplementary Methods for details of taxonomic IDs). After pooling samples into ‘inner’ versus ‘outer’ sites, sample sizes increased to 7–10 measurements per species per location. Subsequent examination of micromorphological features of *Goniastrea* fragments in the laboratory revealed that the specimens represented two separate species that each crossed the gradient (*G*. cf. *favulus* and *G*. cf. *retiformis*) but had limited sample sizes across locations. *Goniastrea* data are therefore used as an intrageneric analysis encompassing intraspecific change in two species.

### Trait data

Physiological, morphological and skeletal trait data were gathered from each individual colony sampled across the gradient using a combination of tissue processing, mass spectrophotometry, scaled image analysis and 3D reconstruction (see Supplementary Methods for details of tissue processing). Zooxanthellae density (ZD), total tissue biomass (TB), protein biomass (PB) and chlorophyll concentration (CH) were quantified from the coral tissue samples. Surface area to volume ratio (SV), branch density (BD), branch width (BW) and corallite width (CW) were quantified from colony planar images and 3D fragment reconstruction. Skeletal density (SD) was measured from the fragments after physiological processing (see Supplementary Methods). We also performed a rudimentary visualisation of changes in colony colour along the gradient using the R‐package ‘colordistance’ (Weller & Westneat, 2019) derived from the photographs of coral colonies (see Supplementary Methods). Traits were selected for analysis because of their role in energy allocation between tissue and skeleton (Anthony et al., 2002), host–symbiont dynamics (Hoogenboom et al., 2015) and colony‐level energy acquisition (McWilliam et al., 2018). The inclusion of different trait types (four tissue, four morphological and one skeletal) also allowed for the comparison of physiological and structural change across the gradient.

### Intraspecific variation

We analysed intraspecific changes in species that occurred within at least one plot inside and outside of the rock islands. The total intraspecific change was analysed using linear mixed effects models of each trait by colony location withf species ID as a random factor. Separate linear models of traits by location were also conducted for each species to quantify differences in intraspecific change. Trait values were log‐transformed and standardised (z‐transformed) to account for different measurement units across traits prior to analysis. Location was modelled as a binary variable from the inside (1) to the outside (0) of the Rock Islands, where outer sites were either barrier or lagoon sites depending on the outer extent of the species distribution. For each linear model, standardised effect sizes of the location of each trait were calculated by dividing model slopes by the standard deviation of the model residuals. An initial Principal component analysis (PCA) of sampled colonies of the seven species that crossed the gradient by their log‐transformed trait values was also conducted to analyse intraspecific trait change.

### Assemblage‐level analysis

Colonies of all 16 sampled species were subsequently used to reconstruct assemblage‐level trait change across the gradient. Three 10 m‐long line intercept transects were used to quantify coral composition and benthic percentage cover within the circular photomosaics at each site. Every colony along the transects was identified to the lowest taxonomic level possible (usually species but sometimes morphospecies), and their intercept (length of transect overlying the colony) was recorded to the nearest centimetre. Sampled species accounted for 72% of total cover on average across transects. The remainder was mostly massive *Porites* (consistently near 15% cover across the gradient), which was not collected, but shows limited trait change across similar gradients (Barkley et al., 2015; Fabricius et al., 2011). Dominant trait values in each assemblage were estimated using community‐weighted means (CWMs), calculated by multiplying site‐level species trait averages by their relative abundance on each transect and summing across species. Linear mixed effects models of CWM values by transect location (inside vs outside the Rock Islands) with site as a random factor were used to quantify standardised effect sizes for each trait.

Intraspecific effects on overall trait change in coral assemblages were measured by calculating differences in ‘fixed’ versus ‘local’ CWMs. Fixed CWMs remove intraspecific effects by using a single (study‐wide) mean for each species, while ‘local’ CWMs incorporate intraspecific effects by using site‐specific species means (Jung et al., 2010). Effect sizes (*E*) of linear mixed models using fixed versus local CWMs for each trait were compared to quantify the influence of species composition (*E*_fixed_) and intraspecific change (*E*_local_ – *E*_fixed_) on assemblage trait structure across locations. Study‐wide assemblage trait variation (within and across locations) caused by intraspecific effects was also verified using sums of squares decomposition of fixed and local CWMs (Lepš et al., 2011). Total sums of squares (*SS*) for composition‐driven variation in CWMs (*SS*_fixed_), intraspecific variation (*SS*_local‐fixed_) and their covariation (*SS*_local_ − *SS*_fixed_ − *SS*_local‐fixed_) were each expressed as a proportion of total assemblage trait variation (*SS*_local_). Species turnover in nine specialist species was also visualised in a secondary PCA of all sampled colonies by log‐transformed trait values.

## RESULTS

### Intraspecific trait change

Every species that crossed the gradient showed consistent shifts in physiological traits in warmer and more acidic locations (Figure 1). Of the 16 species sampled, seven species crossed from typical offshore barrier reefs into the inner Rock Island sites with high temperature, low pH and elevated seawater chlorophyll (Figure 1a,b). They included members of five phylogenetically distant coral genera (*Acropora*, *Isopora*, *Pocillopora*, *Porites* and *Goniastrea*) from four families representing both complex and robust clades of the Scleractinia. Analysis of intraspecific variation in nine traits revealed that species crossing the gradient had highly divergent morphologies and were separated along a single morphological axis from thick lobes and large corallites (e.g. *Goniastrea*) to dense branch clusters and high surface areas (e.g. *Acropora*). In contrast, each species occupied relatively similar positions along an independent axis of tissue and skeletal variation, which was the primary source of intraspecific change as populations traversed the gradient (Figure 1c,d, Figure S1).

**FIGURE 2:**
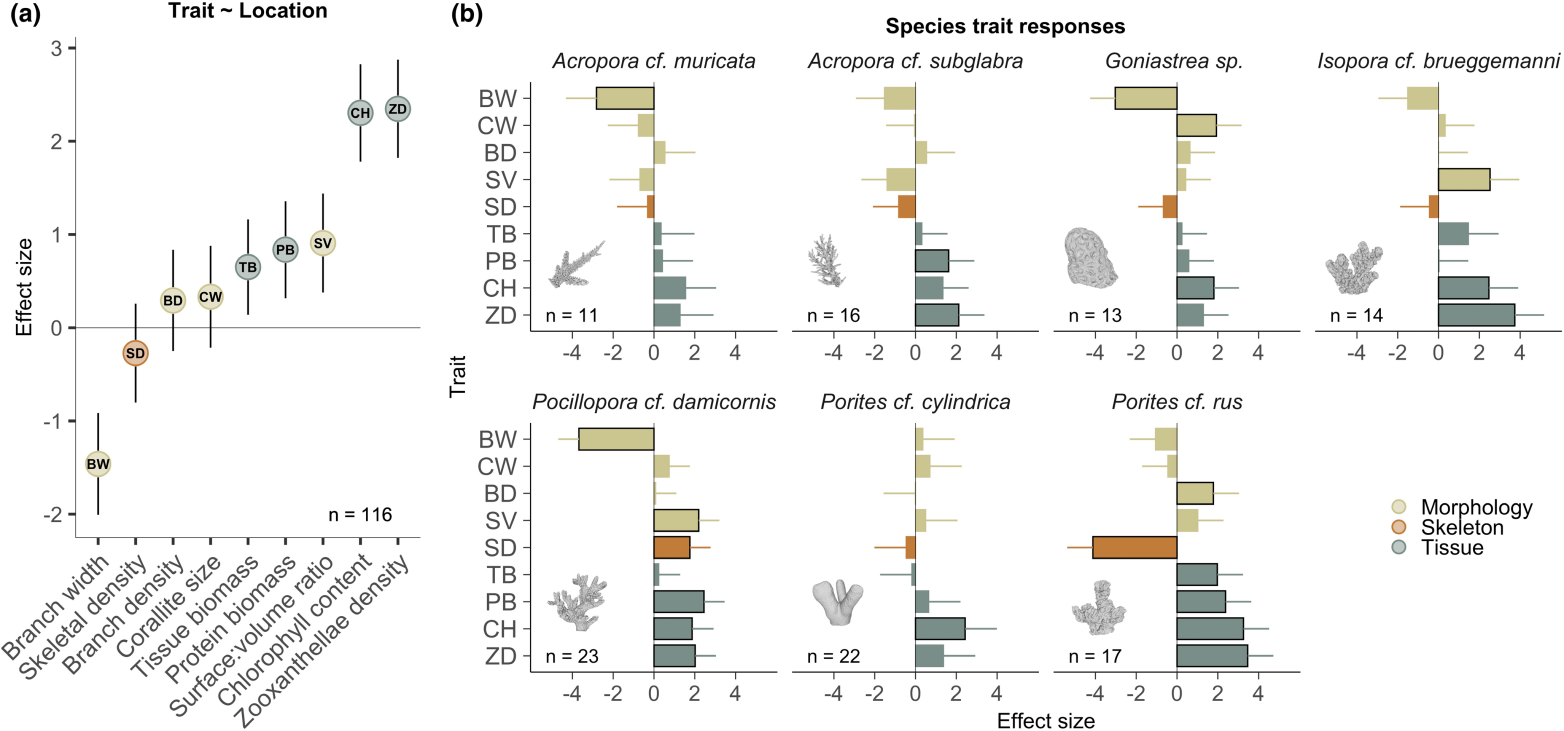
Intraspecific shifts in tissue, skeleton and morphology in warmer, acidified locations. (a) Effect size for colony location (inner versus outer) on nine traits based on a linear mixed effects model. Positive values indicate a trait increase from outer reefs to inner Rock Island sites. (b) Effect size for colony location for nine traits based on separate linear models for each species. Black outlines indicate statistically significant changes. Examples of, 3D models are shown for each species alongside their sample sizes.

Increases in the mass and concentration of tissue components occurred consistently in all species as they crossed into warmer and more acidic sites. However, the exact traits that changed, and the size and significance of these changes were species‐specific (Figure 2, Figure S2). Linear mixed effects models of each trait by colony location for all species combined indicated that four tissue traits show strong and consistent changes across the gradient (Figure 2a; Table S2). Chlorophyll concentrations, symbiont densities, protein biomass and tissue biomass (measured per unit colony area) each showed increases in one or more species at inner reef sites compared with outer (lagoon and barrier) sites, with the largest effect sizes across species (though statistical significance may be affected by sample sizes in individual species models, Figure 2b). Intraspecific changes in morphology and skeleton were more variable across species. For example, many components of colony structure, including corallite width and branch density, did not show strong changes across the gradient (Figure 2, Figure S2). Nevertheless, skeletal density mostly decreased (with the exception of *Pocillopora damicornis*) and there was a clear signal of decreasing branch width (or lobe width in *Goniastrea*) and increasing surface to volume ratios (Figure 2, Figure S2). Skeletal investments (the size and density of skeleton) were therefore mostly reduced as the concentrations of tissue components increased at high‐temperature, low‐pH sites, highlighting a trade‐off between tissue and skeleton as species transitioned into more extreme environments.

**FIGURE 3:**
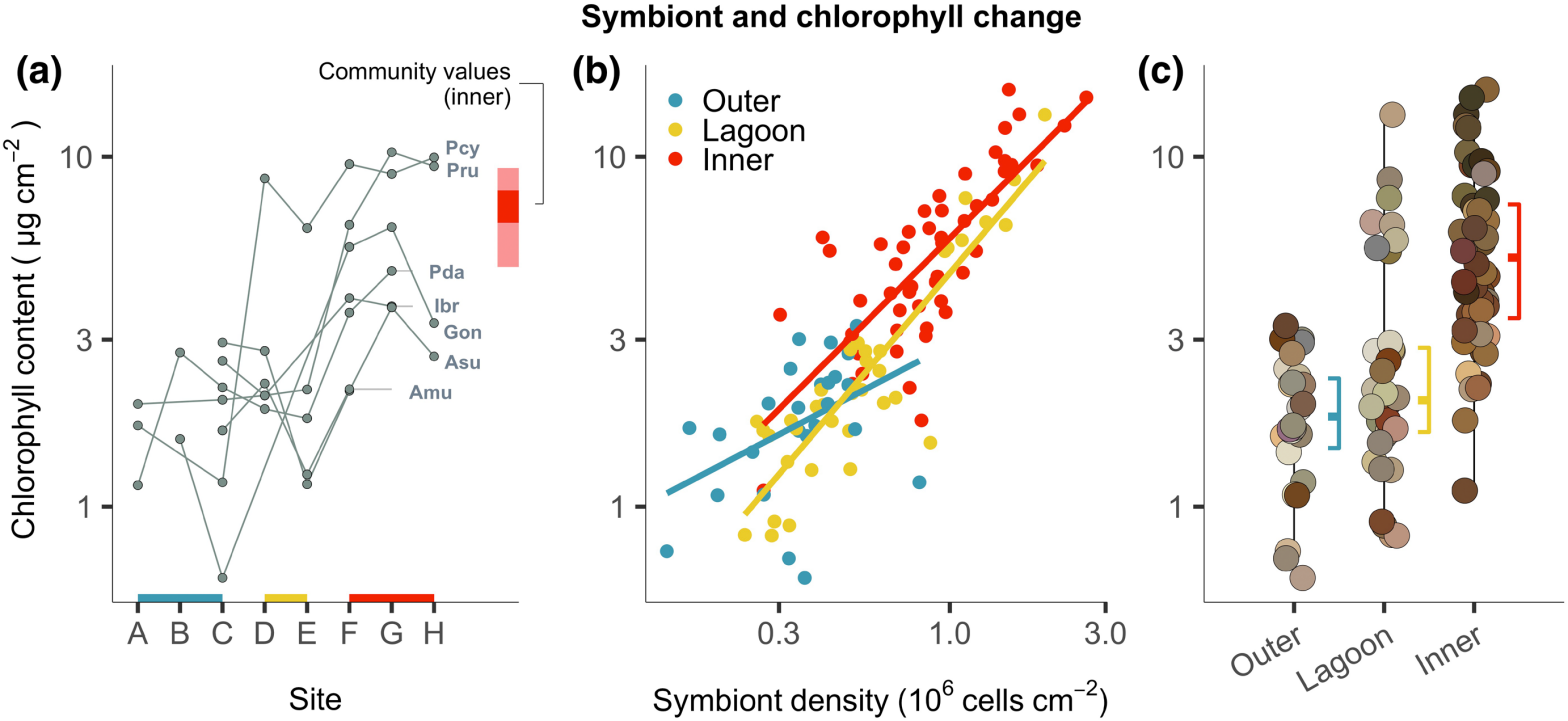
Intraspecific shifts in symbiont traits across the gradient. (a) Shifts in chlorophyll concentrations in seven species that crossed the gradient. The red bar shows the community weighted means of chlorophyll values across inner sites (confidence interval and range), including all species that did not cross the gradient. (b) The relationship between zooxanthellae density and chlorophyll concentrations in three locations. (c) Changes in colony chlorophyll content across three locations (boxplots), with points coloured by an estimate of the dominant colour of the colony. Continuous axes are on a log‐scale.

Symbiont traits showed the largest and most consistent changes as populations traversed the gradient (Figures 2 and 3). Moderate to large increases in mean chlorophyll concentrations (twofold to tenfold increases across species) were closely linked with increases in mean zooxanthellae density (Figure 3a,b), which increased from a few hundred thousand to over 1 million cells/cm^2^ (twofold to fourfold increases across species), and generated strong changes in colony colour (Figure 3c). Although chlorophyll concentrations increased proportionally with symbiont number (log–log slope = 1.03), their relationship changed across locations driven first by an increases in symbiont number, and subsequently by an increase in chlorophyll per symbiont cell (Figure 3b). In contrast to symbiont traits, increases in host tissue traits (tissue and protein biomass) were most pronounced in three species (*Acropora* cf. *subglabra*, *Porites* cf. *rus* and *Pocillopora damicornis*) and were moderate in the other four (Figure 2b). Physiological shifts were therefore disproportionately weighted towards the symbionts as colonies encountered extreme conditions.

### Assemblage trait change

**FIGURE 4:**
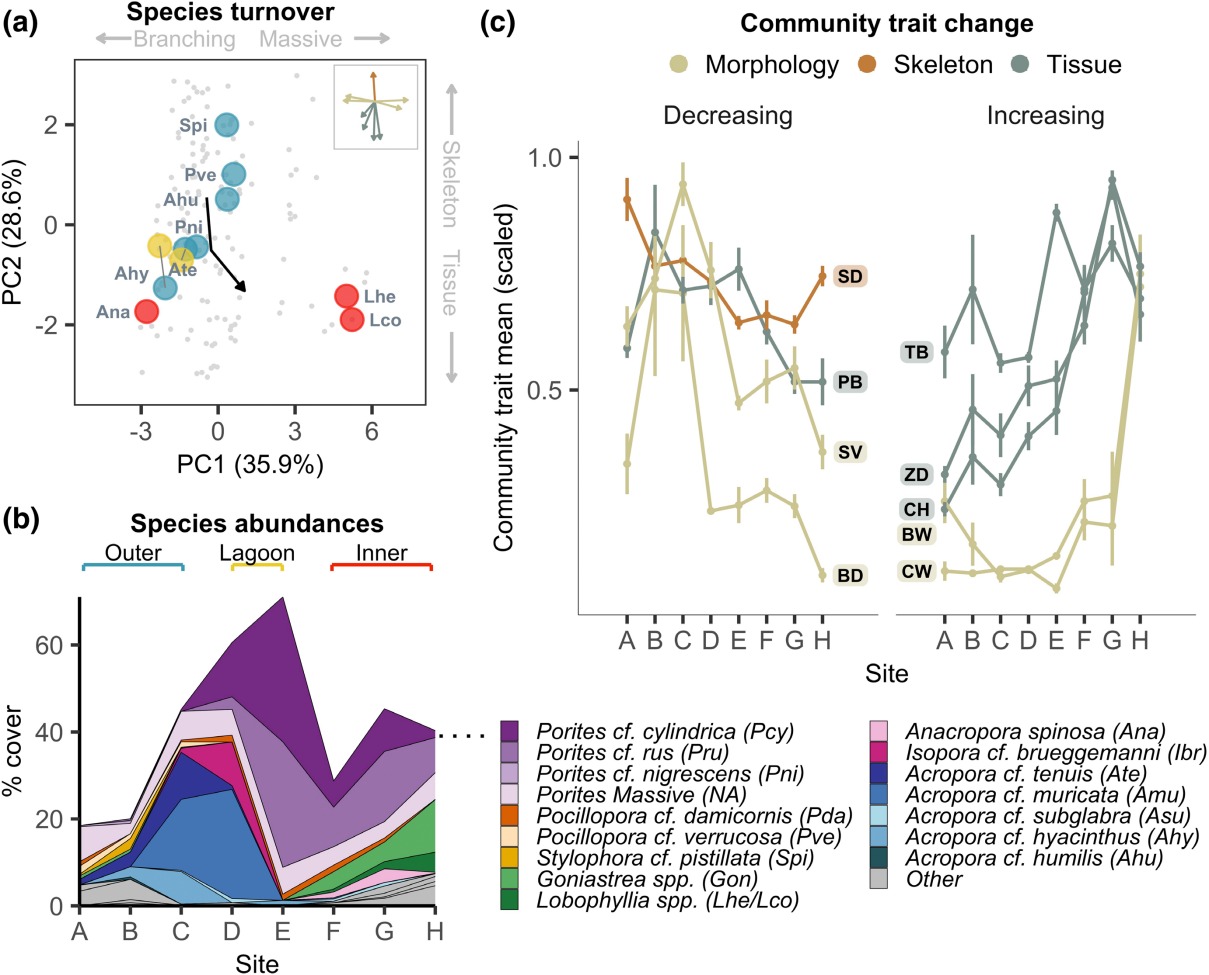
Assemblage‐level shifts in species and trait composition across the gradient. (a) principal components analysis (PCA) of all sampled colonies by nine traits (grey), showing only the average PCA coordinates for eight site‐restricted species that were common in the study system but did not cross the gradient (coloured by location). Trait vectors are shown in the inset. The dark arrow shows the abundance‐weighted shift in PCA coordinates from outer to inner sites. (b) Mean abundances of Scleractinian corals across sites (grey colours = uncollected taxa). (c) Changes in nine community‐weighted trait means (CWMs) across the gradient. Mean and standard error across transects are shown for each CWM at each site. CWMs were scaled for comparability by dividing each value by the maximum transect‐level CWM observed in the study.

Shifts in species composition along the gradient were strongly influenced by physiological differences between species. Nine sampled species that were common in the study system did not cross the gradient but were instead locally dominant at either outer or inner reef sites (Figure 4a). Six common species from four genera (*Porites*, *Stylophora, Pocillopora* and *Acropora*) were restricted to outer barrier and lagoon sites, while three common species from two genera (*Lobophyllia* and *Anacropora*) were restricted to the inner Rock Islands (Figure 4a,b). We sampled the traits of these site‐restricted species and found that trait change driven by species turnover followed a similar trajectory to that of intraspecific change (Figure 1c,d), as species restricted to warmer and acidified sites showed comparatively higher tissue traits and lower skeletal density (Figure 4a). Furthermore, species restricted to the inner Rock Islands with higher tissue concentrations occupied opposite ends of the morphological spectrum, from thick, fleshy lobes (*Lobophyllia*) to fine, fragile branches (*Anacropora*), indicating consistent, interspecific adaptations to environmental stress across a range of morphological types.

Combining all sampled species, we find that intraspecific change and changes in species composition generated substantial shifts in assemblage‐level morphology and physiology across the gradient (Figure 4, Table S3). Diverse communities with high coral cover persisted across sites. Nevertheless, most species increased or decreased in abundance as assemblages shifted from *Acropora*‐dominated reefs, towards *Porites* and *Goniastrea*‐dominated reefs (Figure 4b). Consequently, community trait means weighted by species abundances (CWMs) showed striking changes along the gradient that were significant in all but one trait (Figure 4c, Table S3). In particular, the three‐dimensional branching structure of communities, underpinned by two traits (branch density and surface area per unit volume), showed strong declines towards inner rock island sites, alongside more minor decreases in mean skeletal density and protein biomass. In contrast, three of the four tissue traits showed consistent and gradual increases across the gradient, alongside a shift to large, fleshy corallites and voluminous skeletons (Figure 4c, Figure S3). Coral assemblages inside and outside the rock islands were therefore morphologically and physiologically distinct as average surface complexity declined and average tissue composition changed at sites of environmental stress.

**FIGURE 5:**
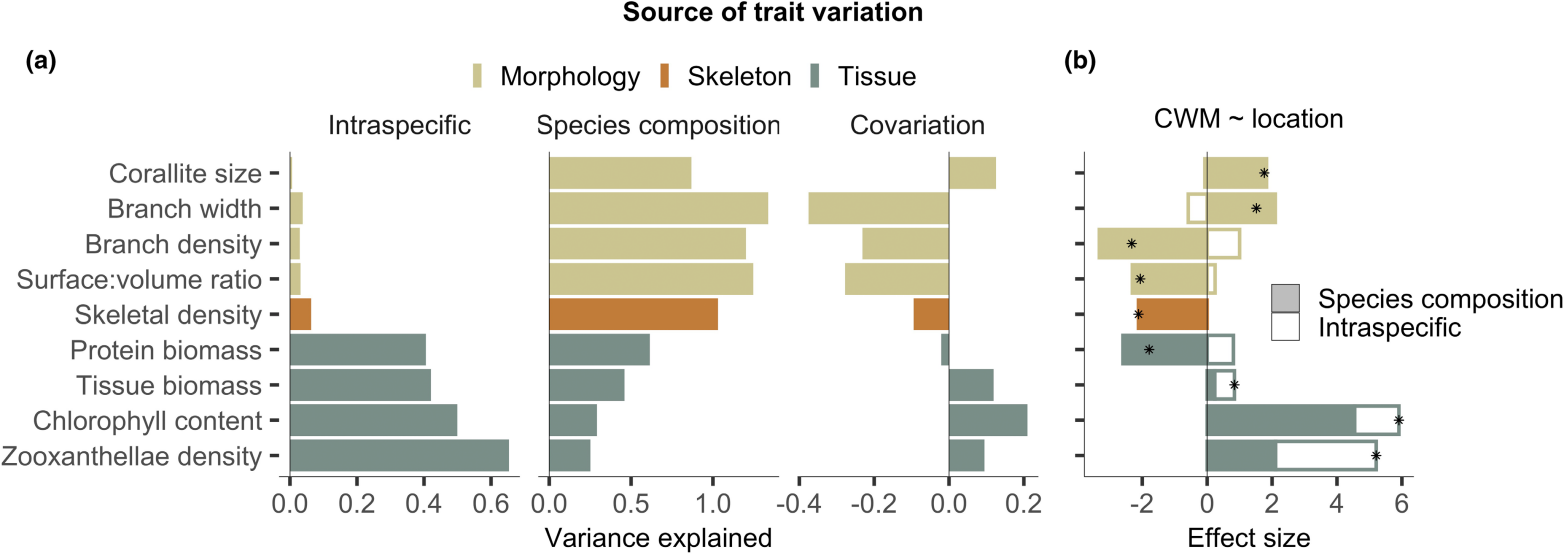
Origins of coral assemblage trait change from optimal to marginal locations. (a) Variation in community trait mean values (CWMs) within and across locations originating from intraspecific differences, changes in species composition and their covariation. (b) Effect sizes for CWM values by assemblage location. Positive and negative values indicate average trait increases and decreases at inner Rock Island sites, respectively. Actual effect sizes for each trait (points) are the net result of shifts in species composition (closed bars) and intraspecific change (open bars). Both analyses are based on differences in fixed versus site‐specific CWMs.

Although changes in both assemblage‐scale morphology and physiology occurred along the gradient, their underlying causes were different (Figure 5). By decomposing study‐wide variation in CWM values into intra‐ versus interspecific change, we find that variation in tissue composition across all study assemblages originated from a relatively even combination (40%–60%) of intraspecific differences and shifts in species composition, while variation in assemblage morphology was driven primarily by species composition (Figure 5a and supplementary Figure S4). Furthermore, intraspecific tissue change drove a large proportion (14%–65%) of CWM change across locations (Figure 5b), increasing the composition‐driven physiological shift by a factor of 1.5 for chlorophyll content and 3 for zooxanthellae densities (positive covariation, Figure 5a,b). In contrast, intraspecific differences in morphology reduced the compositional shift to lower surface area and branch density by 22% and 28%, respectively (negative covariation, Figure 5a,b). Consequently, as populations crossed from outside to inside the Rock Islands, they shifted to match the physiological traits of the dominant species, while intraspecific changes in morphology countered the overall shift towards simpler, mound‐like structures.

## DISCUSSION

This study revealed acute changes in physiological traits in response to a warming, acidifying gradient at the species and assemblage levels. Phylogenetically distant species of reef‐building corals with a diverse range of morphologies consistently showed greater symbiont numbers, higher biomass and reduced skeletal volume and density across the gradient from optimal to extreme conditions. These intraspecific changes combined additively with shifts in species composition to produce physiologically distinct coral communities. Colonies growing in extreme environmental conditions were either habitat specialists with naturally high tissue thickness and symbiont density (e.g. *Lobophyllia*, *Anacropora*), or they were widespread species that shifted away from their ‘typical’ physiology to favour tissue at the expense of skeleton (e.g. *Porites*, *Pocillopora*, *Isopora*, *Acropora*). Directional trait change driven by two processes (species turnover and intraspecific change) provides strong evidence for environmental filtering on colony physiology and suggests that a specific set of physiological traits are ultimately needed for colonies to survive under elevated temperature and acidity. These results underscore the predictive importance of quantifying trait variation both within and among species to anticipate the assemblage‐level impacts of environmental change.

Intraspecific shifts in coral tissue found here reinforce previous observations that long‐term exposure to warming can have complex effects on coral physiology, driven either by genetic change, phenotypic plasticity or both. Across the gradient, we find evidence of reduced calcification (Crook et al., 2013; Strahl et al., 2015), altered host–symbiont dynamics (Morris et al., 2019; Tremblay et al., 2016) and adaptive shifts in chlorophyll content within *Symbiodinum* cells (Howells et al., 2012), while structural changes to skeletal morphology were comparatively minor and inconsistent (Anthony et al., 2002). These consistent intraspecific changes suggest that Rock Island species are adapting or acclimating to warm, acidic conditions, leading to increased fitness and performance in Rock Island communities compared with colonies from the more optimal environment (Barkley et al., 2017; Kurihara et al., 2021). Many of the physiological changes observed are known to occur rapidly due to physiological plasticity (Barott et al., 2021), and the recent, mid‐Holocene development of the Rock Islands may have selected for species with an inherent capability for short‐term acclimation. However, the long residence time of seawater is known to promote genetic differentiation in Palauan corals (Golbuu et al., 2016; Rivera et al., 2022), including locally adapted symbiont populations (Hoadley et al., 2021), and this genetic divergence can enhance intraspecific change and adaptation in corals across small distances (Palumbi et al., 2014; Teixidó et al., 2020). Intraspecific shifts in our analysis are therefore likely to arise from a mix of genetic change and phenotypic plasticity, leading to increased fitness and performance under stress.

Physiological shifts in our analysis can be attributed to altered resource exchange between the host and algal symbionts, which can elevate coral thermal tolerance. Current models of the breakdown of the coral‐algal symbiosis under heat stress involve shifts towards carbon‐limited nutritional dynamics between the partners, triggered by higher metabolic rates, symbiont population growth and reduced translocation of photosynthetic carbon to the host (Morris et al., 2019; Rädecker et al., 2021; Tremblay et al., 2016). Higher symbiont densities and increased chlorophyll at high‐temperature sites suggest increased diversion of resources to the symbionts (Hoogenboom et al., 2015), which can enhance susceptibility to bleaching (Cunning & Baker, 2013). However, coinciding increases in tissue mass and protein content (Figures 2 and 3) suggest that hosts have also increased their capacity to maintain carbon stocks, either through conservative resource‐use strategies or heterotrophic feeding (Wooldridge, 2014), which can facilitate survival under high temperatures (Grottoli et al., 2006). Indeed, similar physiological shifts can reduce bleaching‐driven mortality (Stimson et al., 2002), indicating that observed trait changes could be responsible for elevated bleaching tolerance in Rock Island communities (van Woesik et al., 2012).

Concurrent increases in seawater chlorophyll with temperature across sites (Figure 1a,b) highlight an increased particulate food availability (Kurihara et al., 2021) and greater potential for heterotrophy in the Rock Islands (Fox et al., 2018). Although inputs of anthropogenic nutrients can augment bleaching susceptibility (Donovan et al., 2020), feeding is often found to enhance thermal tolerance, and balanced nutrient supplies can increase rather than diminish coral performance and bleaching resistance (Becker et al., 2021; Edmunds, 2011; Grottoli et al., 2006; Tremblay et al., 2016). Specifically, greater feeding rates can disproportionately enhance symbiont growth while also benefiting host physiology and metabolism, thereby elevating thermal tolerance (Houlbreque & Ferrier‐Pages, 2009; Strahl et al., 2015). Widespread increases in host and symbiont tissue components observed here are therefore consistent with increased nutrient supplies and greater heterotrophic feeding at high‐temperature, low‐pH sites (Hoogenboom et al., 2015). Moreover, at these shallow (~1 m) depths, we find limited evidence of changing turbidity and light levels across the gradient (Figure 1b), suggesting that lower light plays a limited role in the thermal resistance of these communities. Instead, a strong possibility is that greater plankton supplies inside the Rock Islands (inferred from seawater chlorophyll, Figure 1b) are facilitating higher rates of heterotrophic feeding, leading to intraspecific shifts in host–symbiont physiology, and suggesting a greater role of rapid acclimation in driving intraspecific change. Our results therefore support the emerging paradigm that temperature and nutrients will each play strong, interacting roles in coral species responses to elevated temperature and acidity (Barkley et al., 2017; Morris et al., 2019).

Assemblage trait patterns across the gradient indicate that intraspecific variation can have a strong influence on reef evolutionary diversity and morphological complexity in thermally stressful environments. Morphological complexity is crucial for coral reef functioning (Graham & Nash, 2013) and is threatened by global warming as three‐dimensional branching species become depleted after bleaching (Hughes et al., 2018). The warmer and more acidic Palauan rock islands supported communities with substantially lower morphological complexity as boulder‐like species became more dominant, and tabular and bushy *Acropora* were lost (Figure 4). Nevertheless, intraspecific shifts towards finer morphologies (thinner, more crowded branches) countered the overall loss of complexity (Figure 5), suggesting that morphology plays a more proximate, yet complicated role in environmental filtering in this system. Moreover, our study suggests that intraspecific variation allows species from a wide spectrum of morphological groups and distantly related phylogenetic clades (separated by hundreds of millions of years) to cross the gradient using similar strategies (Figure 1c), thereby alleviating the disproportionate loss of genera and families with complex, branching morphologies (Guest et al., 2012). Intraspecific change can therefore limit taxonomic turnover and sustain morphological variation under warm, acidic conditions.

Thermal mass bleaching events are already threatening the long‐term persistence of reef assemblages (Hughes et al., 2018), and reef responses are strongly influenced by species composition, local environmental conditions and history of temperature exposure (van Woesik et al., 2012; Wall et al., 2021). Developing tools to project ecological dynamics under the complex impacts of climate change therefore requires examination of many interacting factors, including a range of traits and their influence on community assembly across environments (Laughlin et al., 2012). For example, plant ecologists recognize that species in arid and semiarid regions tend to have leathery leaves with specific physiological traits, driven by a combination of rainfall and temperature (Wright et al., 2005). Plasticity in these traits can enhance species performance under climatic stress (Liancourt et al., 2015) and enable them to pass through the abiotic ‘filters’ caused by climate change (Jung et al., 2010). Nevertheless, the same traits show opposing responses to climate depending on environmental context (Li et al., 2015; Soudzilovskaia et al., 2013), and spatial gradients do not reliably translate into temporal change (Bjorkman et al., 2018). Although higher tissue and symbiont investments conferred greater fitness to corals under temperature and pH stress, the nutrient‐rich and sheltered bays of Palau may promote intraspecific change that may not otherwise occur. In other circumstances, the interacting effects of local conditions (e.g., nutrients, storms) may alter the influence of traits on species persistence, and limit adaptive plasticity to a comparatively smaller pool of species.

## CONCLUSIONS

Ecological assemblages are increasingly affected by novel or extreme climatic conditions. Our study suggests that the impact of environmental change on assemblages depends on the traits that facilitate persistence and how they vary within and across species. We find that traits related to host‐symbiont tissue physiology are the primary drivers of coral colony establishment across a steep gradient, either through adaptation or phenotypic plasticity. These critical ‘response’ traits were highly variable within species and were loosely associated with morphology, allowing distantly‐related species with varied morphologies to pass through an environmental filter into sites of elevated temperature and acidity. Intraspecific flexibility did not prevent dramatic shifts in species and trait composition (Figure 4b,c). Nevertheless, intraspecific variation ‘buffered’ against the loss of evolutionary and morphological diversity while accelerating shifts in tissue composition. Widespread intraspecific trait changes are likely to be a result of locally heightened nutrient concentrations, which interact with temperature and pH stressors to induce physiological responses. Understanding how the variety of oceanic environments promote or diminish flexibility in key functional traits will therefore be central to understand the assemblages that can persist under ocean warming and acidification.

## AUTHOR CONTRIBUTIONS

MM and TB designed the research and conducted fieldwork. MM, JM, MH, TC and TB conducted laboratory work, data processing and analysis. MM wrote the paper with input from all authors.

### OPEN RESEARCH BADGES

This article has earned Open Data and Open Materials badges. Data and materials are available at https://doi.org/10.5281/zenodo.7075100.

### PEER REVIEW

The peer review history for this article is available at https://publons.com/publon/10.1111/ele.14114.

## Supporting information


Data S1

## Data Availability

Data and code for this study are available for download at https://doi.org/10.5281/zenodo.7075100.
