# Supplementary material for: Intraspecific variation reshapes coral assemblages under elevated temperature and acidity
Source: Ecol Lett. 2022 Oct 9;25(11):2513–24. doi: 10.1111/ele.14114 (PMC9828647; doi:10.1111/ele.14114)
Supplement: Supplementary file 1 — Data S1 [file ELE-25-2513-s001.pdf]

Intraspecific variation reshapes coral assemblages under elevated temperature and acidity

Mike McWilliam, Joshua S. Madin, Tory J. Chase, Mia O. Hoogenboom, Tom C.L. Bridge

Supplementary methods

Taxonomic Identifications: In recent years, molecular phylogenetic data has driven an major changes in coral taxonomy, and considerable uncertainty still exists at the species level, particularly for diverse and ecologically dominant coral genera such as *Porites* and *Acropora* (Cowman et al., 2020). We carefully examined each specimen in the field and the associated skeletal voucher specimens, and used detailed knowledge of the most recent coral taxonomic research to ensure that we collected representatives of distinct species. Yet, we cannot rule out the potential for cryptic speciation within morphological species (Bongaerts et al., 2021). In addition, ongoing molecular taxonomic research suggests that many species are likely undescribed species considered junior synonyms by recent taxonomic revisions based on morphology (Cowman et al., 2020). For example, *Porites rus* is likely to be *Porites iwayamaensis* Eguchi 1935, currently considered a junior synonym of *P. rus*. To minimise confusion, we use species names that conform to the International Code for Zoological Nomenclature, but use the qualifier ‘cf.’ to denote nominal species who’s names are likely to change based on integrated morphological and molecular analysis. To encourage transparency and repeatability in light of this uncertainty, we present details of ongoing revisions, including potential alternative identifications based on the type localities of junior synonyms, including photographs of our species in the field (Supplementary Table 2), and provide skeletal registration vouchers of all samples deposited in the Museum of Tropical Queensland, Australia (Supplementary File 1).

Environmental conditions: Environmental readings from each site (Figure 1b) were taken within a 4-5 hour window (typically 9:00 - 14:00 hours) on a single day using a Eureka Water Probes Manta+ 35A water quality meter (Austin, Texas, USA). Metrics included temperature (accuracy: 0.1 °C), pH (accuracy: 0.1 units), salinity (accuracy: 0.2 PSS), dissolved oxygen (accuracy: 0.1 mg/L), turbidity (accuracy: 0.5 FNU), and chlorophyll concentrations

(accuracy corresponds to a linearity of 0.99 R^2). Replicate readings were taken in October 2019, November 2019, January 2019 and April 2020. The instrument was deployed by hand from a boat, logging profile readings at the desired depth after a one minute pause to allow for sensor stabilization. All sensors are calibrated prior to use on the same day with high quality standard solutions. pH sensors were calibrated using pH-7 and pH-10 buffers, and are on an NBS scale, which prior analysis suggests is likely to be consistently higher than measurements on a total pH scale by approximately 0.1 using this sensor. Long-term temperature averages (between 2011 and 2021) were quantified using the National Oceanic and Atmospheric Administration's coastal watch program to visualise the thermal gradient (Figure 1a). Yet localised readings (Figure 1b) are likely to reflect more accurate temperature information specific to our sites and depths (see also, Colin 2018).

Physiological and morphological analysis: For each sample, physiological analysis was conducted by removing the tissue from the intact skeleton using compressed air, mixing with 15 ml of 0.45 μm-filtered seawater, and homogenising (IKA T10 basic, Ultra Turrax Homogenizer) tissues. Algal symbionts (family: *Symbiodiniaceae*), or zooxanthellae density (ZD) was measured by adding 0.1 ml of formaldehyde to an 0.9 ml aliquot of tissue suspension, counting the cells in six replicate 0.1μl subsamples using a Neubauer Haemocytometer at x40 magnification, and averaging across replicates (Stimson & Kinzie 1991). Chlorophyll was extracted from an 6 ml aliquot of tissue suspension after pelleting symbionts via centrifugation and addition to 4 ml of acetone to extract pigments, allowing them to soak for 12 hours under refrigerated conditions. Chlorophyll concentrations (CC) were measured using spectrophotometry on a SpectraMax Plus^{384} Microplate reader (Molecular Devices). Chlorophyll-a was calculated as $11.43(A_{663} – A_{750}) – 0.64(A_{630} – A_{750})$ and Chlorophyll-c as $-3.63(A_{663} – A_{750}) + 27.09(A_{630} – A_{750})$ where A_n signifies absorbance at wavelength n (Jeffrey & Humphrey 1975). Protein biomass (PB) was quantified from 1ml of tissue using spectrophotometry and the Red 660 protein assay, using bovine serum albumen protein (BSA) to construct a standard curve (Palmer *et al.* 2009). Tissue biomass (TB) measuring the total organic weight of the tissue (ash-free dry weight) was quantified by placing 5ml of tissue suspension into a freeze dryer (Christ, Alpaa 1-1 LO plus) for 48 hours to calculate dry weight, and then subtracting the ash weight measured after incinerating the sample in a muffle furnace at 550°C (Leuven & Brock 1985).

Tissue surface area, skeletal volume, and surface area to volume ratio (SV) of each fragment was measured to 0.1mm resolution using a 3D laser scanner (CREAFORM HandySCAN 3D and VXelements software). Physiological traits were scaled to coral tissue area by calculating total content per fragment accounting for the initial 15 ml dilution of water, and dividing by fragment area. The skeletal density (SD) of fragments was found by dividing the dry fragment weight (to the nearest 0.1g) by its volume. Morphological parameters such as planar surface area, corallite width, and branching dimensions were quantified from images using ImageJ (version 1.53a). Projections of the colony greater than 3cm in length were considered a new branch, excluding individual corallites in bottlebrush species, and classifying individual lobes as branches for mound-shaped species. Branch density (BD) was measured by dividing the total number branches per colony by planar area. Branch width (BW) was measured as the average diameter of three randomly selected branches measured halfway between the branch tip and the branch base. Branch height (BH) was measured as the average perpendicular length of three randomly selected branches. Finally, we used the R-package 'colordistance' (Weller & Westneat, 2019) to generate a rudimentary visualisation of colony colours across the gradient (shown in Figure 3C only). We took a planar image of the colony in broad daylight using a Canon G7X, took a 3 cm^2 snapshot of the centre of each colony, and used 'colordistance' to identify the dominant colour (most frequent RGB bin across pixels) for each image.

Methods references

Bongaerts, P., Cooke, I. R., Ying, H., Wels, D., den Haan, S., Hernandez-Agreda, A., Brunner, C. A., Dove, S., Englebert, N., Eyal, G., Forêt, S., Grinblat, M., Hay, K. B., Harii, S., Hayward, D. C., Lin, Y., Mihaljević, M., Moya, A., Muir, P., … Hoegh-Guldberg, O. (2021). Morphological stasis masks ecologically divergent coral species on tropical reefs. Current Biology, 31(11), 2286-2298.e8.

Colin, P. (2018). Ocean Warming and the Reefs of Palau. Oceanography, 31(2). https://doi.org/10.5670/oceanog.2018.214

Cowman, P. F., Quattrini, A. M., Bridge, T. C. L., Watkins-Colwell, G. J., Fadli, N., Grinblat, M., Roberts, T. E., McFadden, C. S., Miller, D. J., & Baird, A. H. (2020). An enhanced target-enrichment bait set for Hexacorallia provides phylogenomic resolution of the staghorn corals (Acroporidae) and close relatives. Molecular Phylogenetics and Evolution, 153, 106944.

Jeffrey SW, Humphrey GF (1975) New spectrophotometric equations for determining chlorophylls *a*, *b*, c_1, and c_2 in higher plants, algae and natural phytoplankton. Biochemie und Physiologie de Pflanzen 167:191-194

Leuven R, Brock TCM, van Druten HAM (1985) Effects of preservation on dry and ash-free dry-weight biomass of some aquatic macro-invertebrates. Hydrobiologia 127: 151-159

Stimson J, Kinzie RA (1991) The temporal pattern and rate of release of zooxanthellae from the reef coral *Pocillopora damicornis* (Linnaeus) under nitrogen-enrichment and control conditions. Journal of Experimental Marine Biology 15:63-74

Palmer CV, Modi CK, Mydlarz LD (2009) Coral fluorescent proteins as antioxidants. PLoS ONE 4: e7298. Doi:10.1371/journal.pone.0007298

Weller, H. I., & Westneat, M. W. (2019). Quantitative color profiling of digital images with earth mover's distance using the R package colordistance. PeerJ, 7, e6398. https://doi.org/10.7717/peerj.6398

Appendix S1

Supplementary table 2

	***Isopora* cf. *brueggemanni* (Ibr)**	***Acropora* cf. *subglabra* (Asu)**	***Porites* cf. *cylindrica* (Pcy)**
	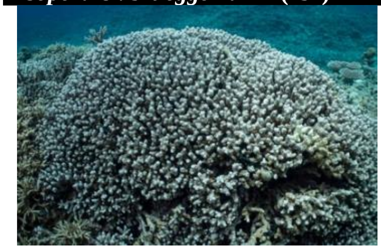	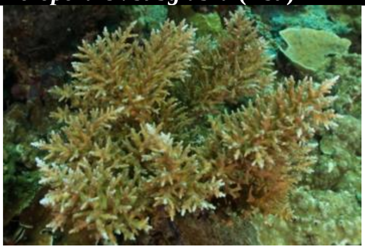	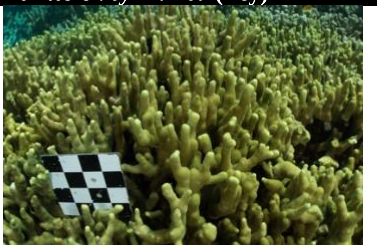
Sampled at:	Inner & Outer Sites	Inner & Outer Sites	Inner & Outer Sites
Authority:	*Madrepora brueggemanni* Brook 1893	*Madrepora subglabra Brook 1891*	*Porites cylindrica* Dana 1846
Type Locality:	Singapore	South Seas	Lizard Island (neotype)
Possible junior synonyms:	*Madrepora pelewensis* Rehberg 1892<br>*Acropora meridiana* Nemenzo 1971	*Acropora spiniformis* Eguchi & Shirai 1977	*Porites planocella* Nemenzo 1955

	***Acropora* cf. *muricata* (Amu)**	***Pocillopora* cf. *damicornis* (Pda)**	***Goniastrea* cf. *retiformis* (Gon)**
	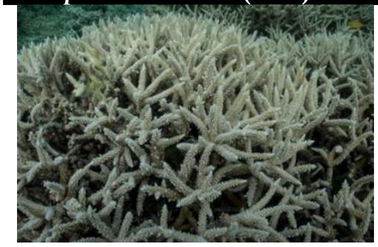	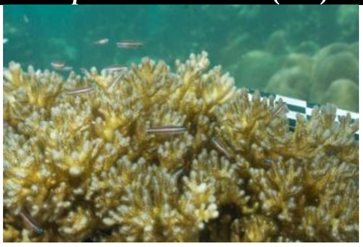	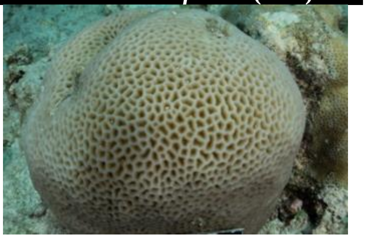
Sampled at:	Inner & Outer Sites	Inner & Outer Sites	Inner & Outer Sites
Authority:	*Millepora muricata Linnaeus 1758*	*Millepora damicornis*, Linnaeus 1758	*Astraea retiformis Lamarck 1816*
Type Locality:	Banda Sea (neotype)		Seychelles
Possible junior synonyms:	Several, most closely resembles *Madrepora brachiata* Dana 1846, from the Sooloo Sea		

	***Porites* cf. *rus* (Pru)**	***Porites* cf. *nigrescens* (Pni)**	***Acropora* cf. *hyacinthus* (Ahy)**
	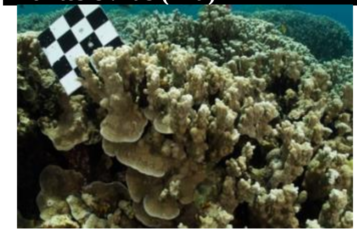	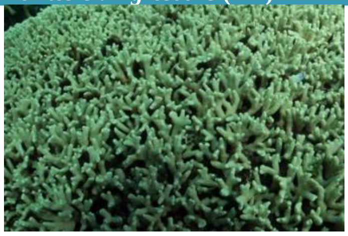	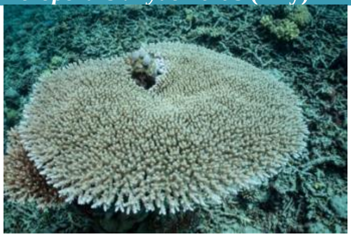
Sampled at:	Inner & Outer Sites	Outer Sites	Outer Sites
Authority:	*Madrepora rus* Forskål 1775	*Porites nigrescens* Dana 1846	*Madrepora hyacinthus* Dana 1846
Type Locality:	Red Sea	Fiji	Fiji
Possible junior synonyms:	*Porites iwayamaensis* Eguchi 1935		*Madrepora pectinata* Brook 1892<br>*Acropora bifurcata* Nemenzo 1971

	***Acropora* cf. *humilis* (Ahu)**	***Stylophora* cf. *pistillata* (Spi)**	***Acropora* cf. *tenuis* (Ate)**
	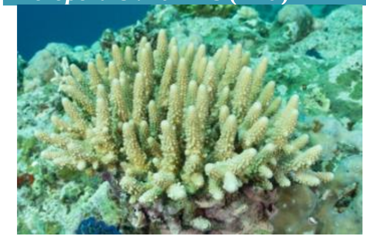	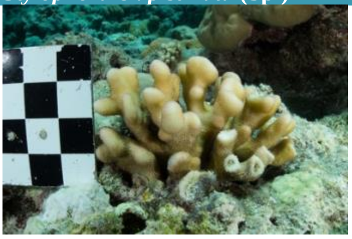	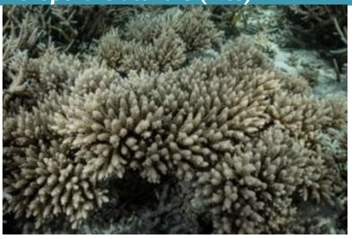
Sampled at:	Outer Sites	Outer Sites	Outer Sites
Authority:	*Madrepora humilis* Dana 1846	*Madrepora pistillata Esper 1792*	*Madrepora tenuis* Dana 1846

Type Locality:	Fiji	Indian Ocean	Fiji
Possible junior synonyms:	No obvious potential candidates	Several, including *Stylophora nana* Nemenzo 1964	*Madrepora bifaria* Brook 1892

	***Pocillopora* cf. *verrucosa* (Pve)**	***Lobophyllia* cf. *corymbose* (Lco)**	***Lobophyllia* cf. *hemprichii* (Lhi)**
	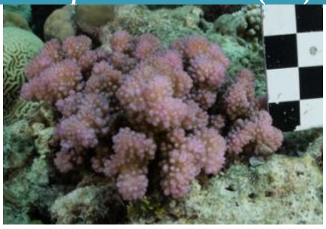	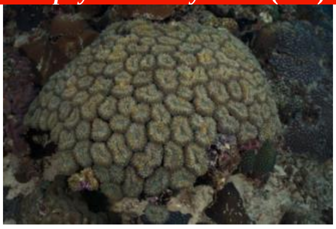	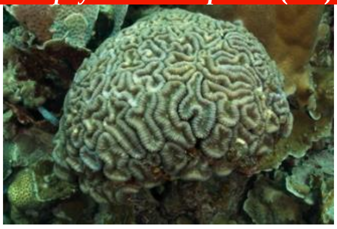
Sampled at:	Outer sites	Inner Sites	Inner Sites
Authority:	*Madrepora verrucosa* Ellis & Solander 1786	*Madrepora corymbosa* Forskål 1775	*Manicina hemprichii* Ehrenberg 1834
Type Locality:	Lizard Island (neotype)	Red Sea	Red Sea
Possible junior synonyms:			

	***Anacropora spinosa* (Ana)**	***Goniastrea* cf. *favulus* (Gfa)**
	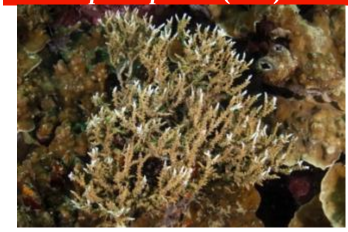	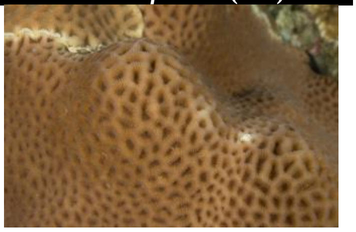
Sampled at:	Inner Sites	Inner Sites
Authority:	*Anacropora spinosa* Rehberg 1892	*Astraea favulus* Dana 1846
Type Locality:	Palau	Fiji
Possible junior synonyms:	None	

Table S2: Taxonomic notes on collected samples.

Appendix S1

Supplementary table 1

Site label	Location	Latitude	Longitude
A	Outer	7.292581°N	134.241208°E
B	Outer	7.260378°N	134.522294°E
C	Outer	7.280271°N	134.527047°E
D	Lagoon	7.306962°N	134.506323°E
E	Lagoon	7.372726°N	134.387753°E
F	Inner	7.32192°N	134.499672°E
G	Inner	7.31919°N	134.489698°E
H	Inner	7.319654°N	134.488449°E

Table S1: Locations of the sampling sites in Palau.

Appendix S1

Supplementary figure 1

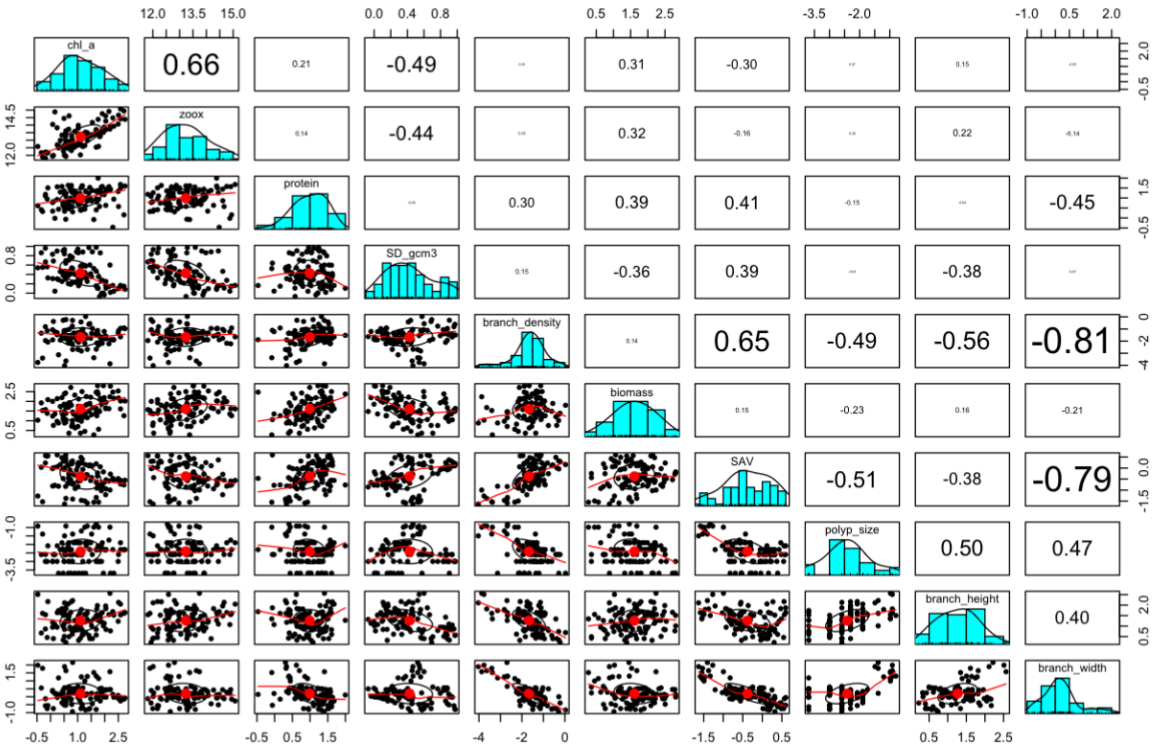

Figure S1: Summary of relationships among traits of sampled colonies. Data is shown in lower panels. Correlation coefficients in upper panels. The plot was produced using the R package “psych”.

Appendix S1

Supplementary Table 2

Trait		Estimate	s.e.	df	t	p	r^2_m	r^2_c
Branch width	Int.	0.43	0.40	7	1.1	0.31	0.069	0.83
	Slope	- 0.66	0.12	70	-5.2	**< 0.001**		
Skeletal density	Int.	- 0.01	0.35	6	-0.02	0.98	0.002	0.86
	Slope	- 0.09	0.09	71	-1.0	0.31		
Branch density	Int.	-0.12	0.38	8	-0.32	0.75	0.006	0.64
	Slope	0.19	0.18	71	1.1	0.29		
Corallite width	Int.	0.003	0.39	7	0.009	0.99	0.004	0.83
	Slope	0.14	0.11	70	1.2	0.23		
Tissue biomass	Int.	-0.50	0.24	20	-2.0	0.051	0.073	0.17
	Slope	0.63	0.25	73	2.5	**0.015**		
Protein biomass	Int.	- 0.54	0.29	9	-1.8	0.099	0.084	0.41
	Slope	0.68	0.21	74	3.2	**0.002**		
Surface area per volume	Int.	-0.24	0.39	6	-0.63	0.551	0.014	0.91
	Slope	0.28	0.08	71	3.4	**0.001**		
Chlorophyll content	Int.	-0.78	0.20	11	-3.9	**0.002**	0.432	0.60
	Slope	1.36	0.15	74	8.6	**< 0.001**		
Zooxanthellae density	Int.	- 0.89	0.20	11	-4.4	**0.001**	0.443	0.61
	Slope	1.42	0.16	75	8.7	**< 0.001**		

Table S2: Intraspecific change across the gradient. Values show the output of linear mixed effects models examining the relationship between location and trait values for species that crossed the gradient. The model included species as a random effect. The r^2 values shown are the marginal r^2 (r^2_m), indicating the variance explained by fixed factors, and conditional r^2 (r^2_c), indicating the variance explained by fixed and random factors.

Appendix S1

Supplementary Table 3

Trait		Estimate	s.e.	df	t	p	r^2_m	r^2_c
Branch density	Int.	0.58	0.30	4	1.89	0.12	0.41	0.65
	Slope	-1.45	0.28	27	-5.27	< **0.001**		
Surface area per volume	Int.	0.60	0.28	4	2.20	0.086	0.40	0.58
	Slope	-1.37	0.29	28	-4.80	< **0.001**		
Skeletal density	Int.	0.44	0.40	4	1.08	0.34	0.27	0.71
	Slope	-1.28	0.27	26	-4.66	< **0.001**		
Protein biomass	Int.	0.66	0.19	28	3.39	**0.002**	0.44	0.44
	Slope	-1.32	0.27	28	-4.80	< **0.001**		
Tissue biomass	Int.	-0.25	0.35	5	-0.72	0.504	0.09	0.42
	Slope	0.66	0.34	27	1.92	0.064		
Corallite width	Int.	-0.58	0.27	7	-2.15	0.071	0.31	0.54
	Slope	1.09	0.26	28	4.06	< **0.001**		
Branch width	Int.	- 0.57	0.23	11	-2.42	**0.034**	0.32	0.40
	Slope	1.12	0.29	29	3.76	< **0.001**		
Zooxanthellae density	Int.	-0.81	0.24	4	-3.2	**0.026**	0.67	0.88
	Slope	1.80	0.15	25	11.4	< **0.001**		
Chlorophyll content	Int.	-0.84	0.22	4	-3.7	**0.018**	0.73	0.90
	Slope	1.91	0.14	25	12.9	< **0.001**		

Table S3: Community trait across the gradient. Values show the output of linear mixed effects models examining the relationship between location and trait values for species that crossed the gradient. The model included species as a random effect. The r^2 values shown are the marginal r^2 (r^2_m), indicating the variance explained by fixed factors, and conditional r^2 (r^2_c), indicating the variance explained by fixed and random factors.

Appendix S1

Supplementary figure 2

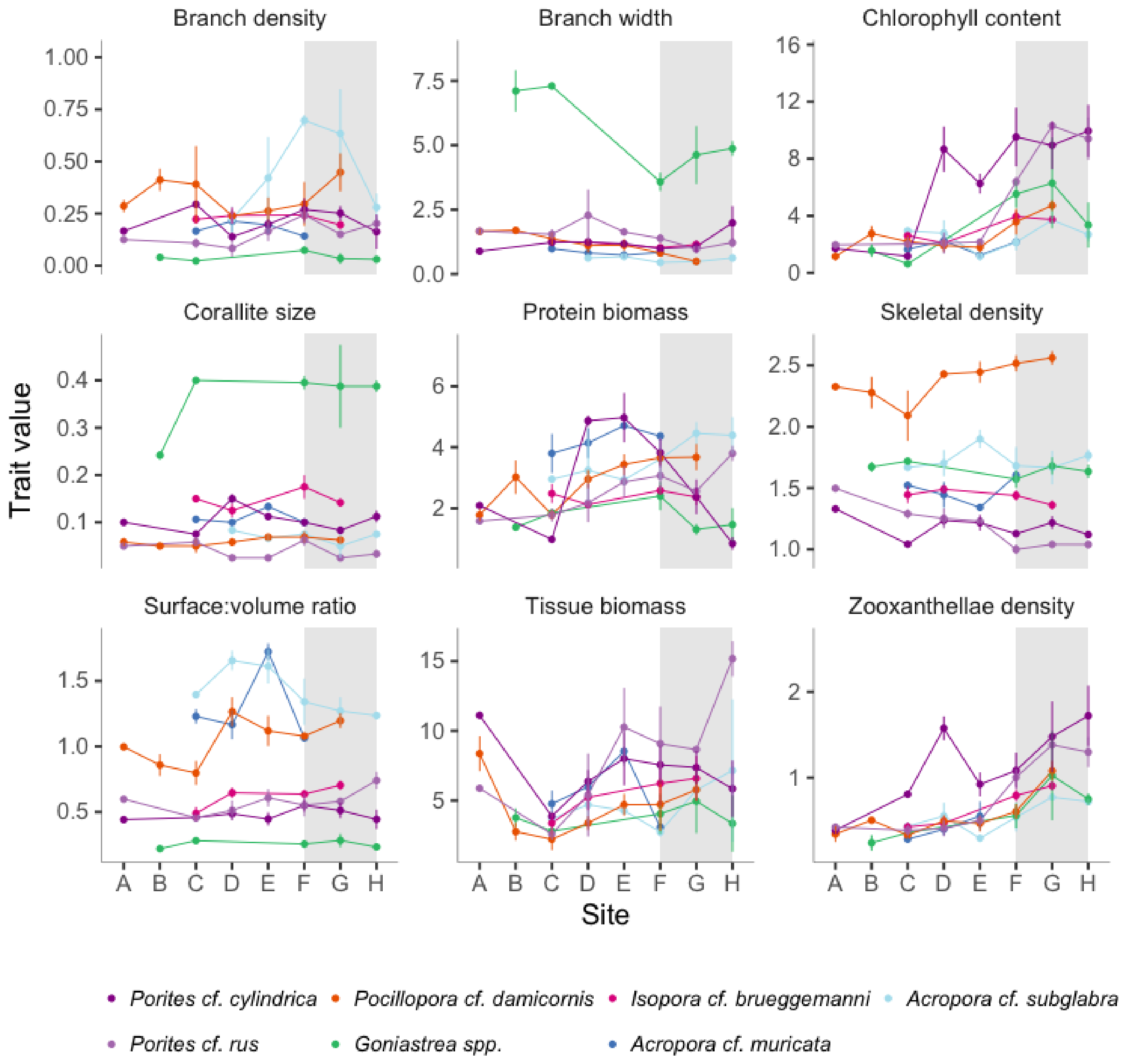

Figure S2: Intraspecific trait change across sites. Each coloured line is one of the seven species that crossed the gradient. Each panel is a trait. Means and standard error bars or each trait at each site are shown. The units of each axis or trait are: µl cm^{-2} (chlorophyll concentration), 10^6 cells cm^{-2} (zooxanthellae density), mg cm^{-2} (protein and tissue biomass), branches cm^{-2} (branch density), cm (corallite and branch width), g cm^{-3}(skeletal density), and cm^2 cm^{-3} (surface to volume ratio). The grey bar indicates sites that were inside the Rock Islands.

Appendix S1

Supplementary figure 3

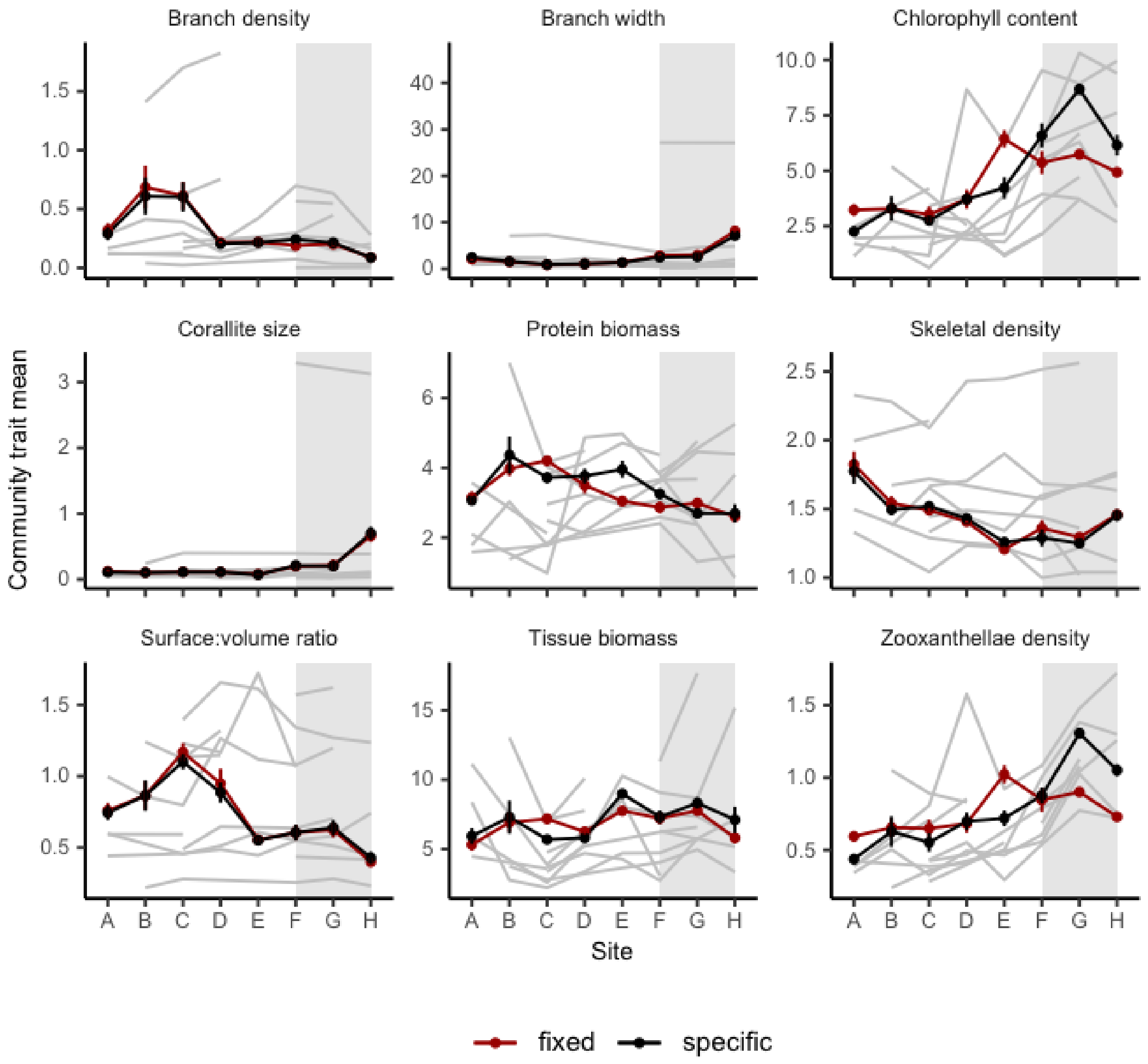

Figure S3: Community trait change across sites. Each panel is a trait. Community means and standard error bars or each trait at each site are shown by the black and red lines. Species means at each site are shown by the grey lines. The units of each axis or trait are: μl cm^{-2} (chlorophyll concentration), 10^6 cells cm^{-2} (zooxanthellae density), mg cm^{-2} (protein and tissue biomass), branches cm^{-2} (branch density), cm (corallite and branch width), g cm^{-3}(skeletal density), and cm^2 cm^{-3} (surface to volume ratio). Fixed CWMs (red) quantify species composition effects only, while specific CWMs (black) quantify species composition plus intraspecific variation. The grey bar indicates sites that were inside the Rock Islands.

Appendix S1

Supplementary figure 4

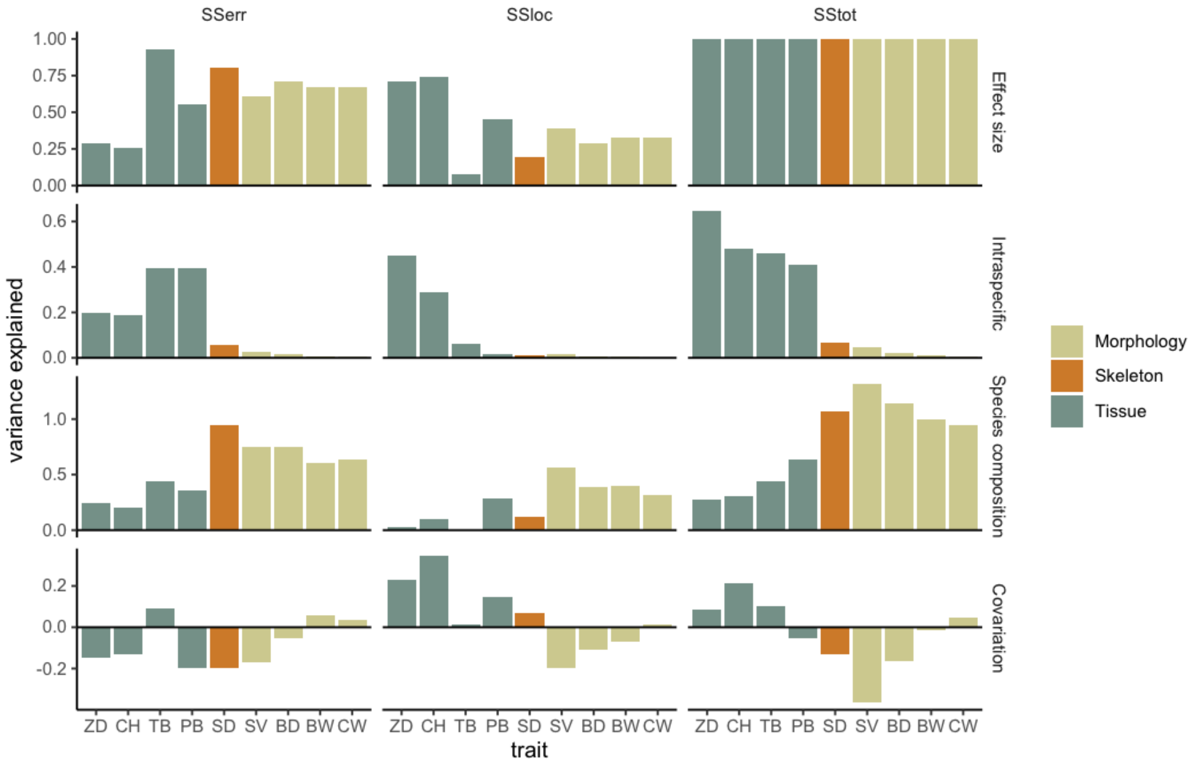

Figure S4: Sums of squares decomposition of the effects of location on community trait means. Columns show the decomposition of variation into within-group variation (SSerr), between-group variation driven by inner versus outer locations (SSloc) and total variation (SStot). Rows show the decomposition of variation into intraspecific and turnover effects calculated using fixed and local means. Following Leps et al (2011), total effect sizes of traits were quantified using ANOVA of local means, the contribution of species turnover was quantified using ANOVA of fixed means, and the contribution of intraspecific variation was quantified using an ANOVA of the difference between fixed and local means. Covariation between the intraspecific variation and species turnover is calculated as the total effect size minus intraspecific and turnover effects. Between group effects (SSloc) are presented in figure 4.
